# Ultrasound-based examination of the medial ligament complex shows gender- and age-related differences in laxity

**DOI:** 10.1007/s00167-020-06293-x

**Published:** 2020-09-23

**Authors:** Patricia M. Lutz, Matthias J. Feucht, Judith Wechselberger, Michael Rasper, Wolf Petersen, Klaus Wörtler, Andreas B. Imhoff, Andrea Achtnich

**Affiliations:** 1 Department for Orthopedic Sports Medicine, Technical University Munich, Ismaninger Str. 22, 81675 Munich, Germany; 2 Department of Orthopedics and Trauma Surgery, Medical Center, Faculty of Medicine, Albert-Ludwigs-University of Freiburg, Freiburg, Germany; 3 Department of Diagnostic and Interventional Radiology, Technical University of Munich, Munich, Germany; 4 Martin-Luther-Hospital, Berlin, Germany

**Keywords:** Ultrasound, MCL, Healthy knee, Laxity, Medial joint space

## Abstract

**Purpose:**

Ultrasound (US) examination of the medial joint space of the knee has played a subordinate diagnostic role up till now. The purpose of the present study was to describe mean values of medial joint width and to investigate the impact of gender, age, and body mass index (BMI) on medial joint laxity in healthy knees using modern, dynamic US in a standardized fashion in unloaded and standardized loaded conditions.

**Methods:**

A total of 65 subjects with 79 healthy knees were enrolled in this study. All volunteers underwent clinical examination of the knee. The medial knee joint width was determined using US in a supine position at 0° and 30° of knee flexion in unloaded and standardized loaded (= 15 Dekanewton, daN) conditions using a specific device. Mean values were described and correlations between medial knee joint width and gender, age, and BMI were assessed.

**Results:**

Thirty-two females and 33 males were enrolled in this study. The mean medial joint width in 0° unloaded was 5.7 ± 1.2 mm and 7.4 ± 1.4 mm loaded. In 30° of knee flexion, the mean medial joint width was 6.1 ± 1.1 mm unloaded and 7.8 ± 1.2 mm loaded. The average change between unloaded and loaded conditions in 0° was 1.7 ± 1.0 mm and in 30° 1.7 ± 0.9 mm. A significant difference between genders was evident for medial joint width in 0° and 30° of flexion in unloaded and loaded conditions (*p* < 0.05). With rising age, a significant increased change of medial joint space width between unloaded and loaded conditions could be demonstrated in 0° (*p* = 0.032). No significant correlation between BMI and medial joint width in US could be found.

**Conclusion:**

Mean values of medial joint width in unloaded and standardized loaded conditions using a fixation device could be demonstrated. Based on the results of this study, medial knee joint width in US is gender- and age-related in healthy knees. These present data may be useful for evaluating patients with acute or chronic pathologies to the medial side of the knee.

**Level of evidence:**

III.

## Introduction

The importance of the medial collateral ligament (MCL) as a primary stabilizer against valgus stress on the knee joint has been proven by numerous biomechanical studies [7, 19, 31]. Furthermore, insufficiency of the medial ligament complex also leads to increased rotational instability in both internal and external rotation [23, 26, 28].

Since the medial ligament complex is frequently affected by injuries [2, 4, 22, 30], research on the medial side of the knee has been centered around pathologic findings on the medial collateral ligament via magnetic resonance images (MRI) and clinical examinations [10, 16, 20]. The current gold standard for clinical evaluation of medial joint space width is the valgus stress test: medial joint space width is then estimated in millimeters (mm) to classify the medial instability [5, 10, 30]. Radiological examination of the medial side of the knee by radiographs using a fixation device [13] was replaced by a static MRI examination [15]. Fluoroscopic imaging of the knee under valgus stress can also be used to assess medial knee instability [13, 25]. In the late 1980s, ultrasound (US) examination had been described to assess medial structures of the knee [6, 21]. In comparison to radiographs, this method is immediately available, avoids radiation, is cheap, and allows for a dynamic assessment [6, 8, 21]. Additionally, a side-to-side comparison is possible. This might be a good alternative, especially using the currently introduced portable ultrasound devices.

At the moment, there are only few studies available investigating laxity of the medial ligament complex by US examination [8, 12, 21, 25, 32]. Existing studies cover small case numbers [12], include cadaveric examinations [25], or use more than 20-year-old US technology [8, 21].

However, in recent years, scientific interest in US as diagnostic tool for examinations of the knee joint has grown again [1, 3, 14, 24].

Physiological values concerning the medial laxity in healthy knees in US examinations via the use of applied valgus stress are missing. Considering diagnosis of frequent ligamentous injuries to the medial side of the knee, US results of this study could help clinicians to better understand and classify acute or chronic pathologies of the medial side of the knee.

The purpose of the present study was to describe mean values of medial joint width and to investigate the impact of gender, age, and BMI on medial joint laxity in healthy knees using modern, dynamic US in a standardized fashion in unloaded and standardized loaded conditions. We hypothesized that laxity is depending on gender and age.

## Materials and methods

The study was approved by the institutional review board of the Technical University of Munich (235/19 S) and conducted according to the Declaration of Helsinki. All subjects gave their written informed consent to participate in this investigation. In this prospective study, medial ligament laxity in healthy knee joints was assessed. Inclusion criteria were: subjects aged > 18 years with no history of knee injuries and knee pain within the last three months. Exclusion criteria were subjective or objective ligament instability, previous knee operations, subjective knee pain or severe leg malalignment. Subgroup analysis concerning gender, age, and BMI was performed.

### Clinical examination

All volunteers underwent clinical examination of the knee. Examination of ligament stability included testing the laxity of the MCL and lateral collateral ligament (LCL), as well as the anterior and posterior cruciate ligament. Meniscus was evaluated by joint space tenderness and through the Steinmann test [27]. Frontal plane knee alignment was determined by clinical measures, similar to the Caliper method, as described in 2006 by Hinman et al*.* [9]. A distance of more than 3 cm (cm) was interpreted as severe leg deformity (medial knee joint line distance or medial malleoli distance). Since only healthy knees were enrolled in this study, using radiographs to determine frontal plane knee alignment would not have been in accordance with ethical standards.

### Radiological evaluation

US examination was performed by two board-certified radiologists with at least 5 years of experience in musculoskeletal imaging at our institution. All acquired images of the knees were evaluated through the picture archiving and communication system PACS workstations (Agfa, Ridgefield Park, NJ, USA).

### Ultrasound (US)

**Fig. 1:**
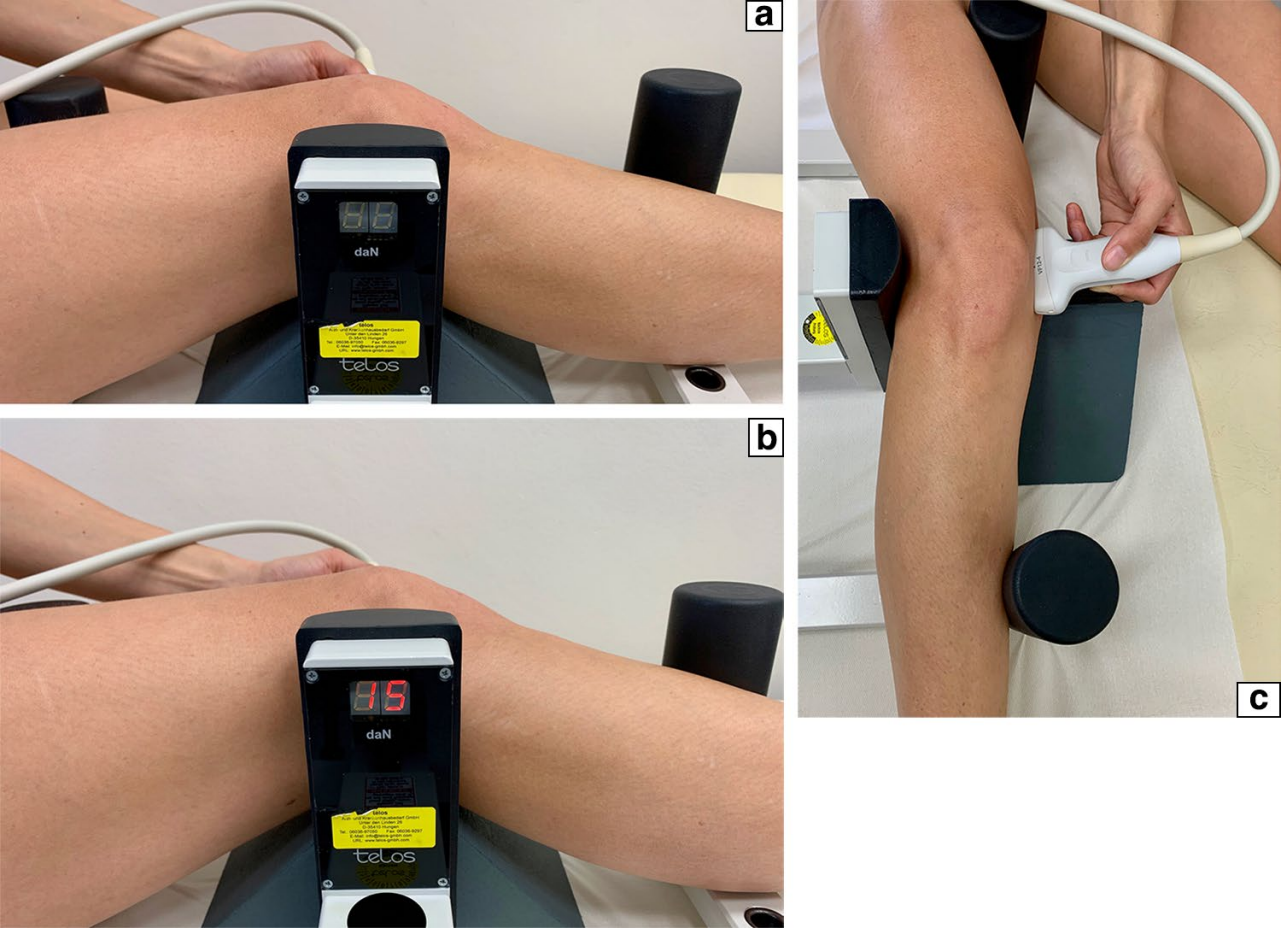
**a** 30° bended knee in TELOS fixation device without load, view from the lateral side; **b** 30° bended knee in Telos fixation device with load (15 daN), view from the lateral side; **c** 30° bended knee in Telos fixation device with load (15 daN), view from the top;

**Fig. 2:**
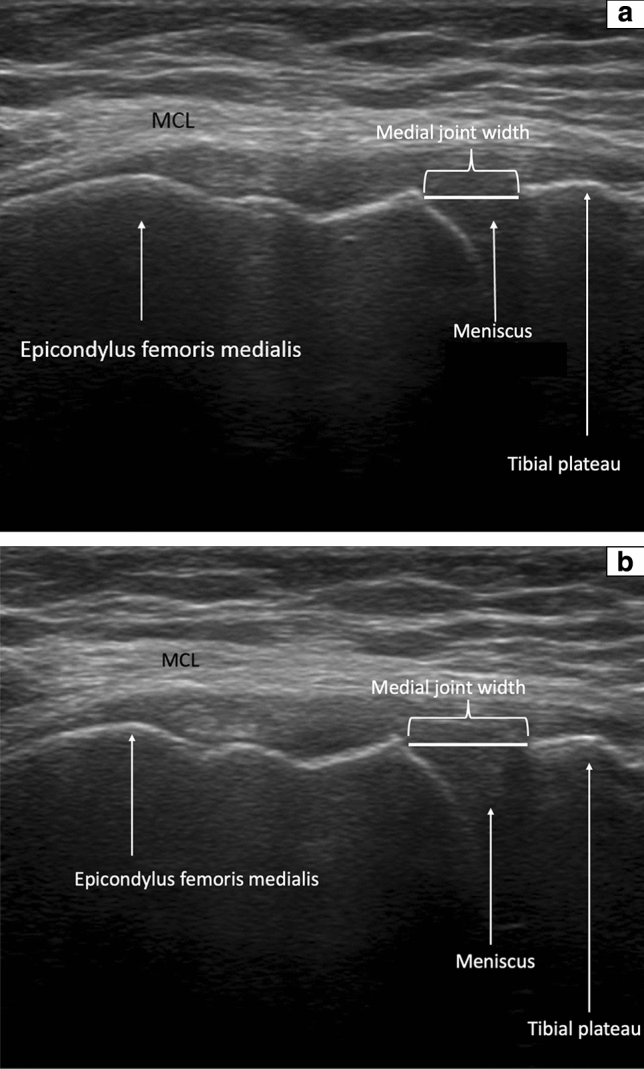
**a** US image of the medial joint width in 30° of knee flexion without TELOS. White arrows show epicondylus femoris medialis, meniscus, and tibial plateau. One white line is positioned between femoral and tibial margins where medial joint width can be measured in mm. **b** US image of the medial joint width in 30° of knee flexion in loaded condition with TELOS fixation device (15 daN). *daN* Dekanewton, *MCL* medial collateral ligament, *mm* millimeter

To evaluate the medial ligament laxity, the medial knee joint width was assessed via the ACUSON NX3 Ultrasound System (Siemens Erlangen, Germany), a linear transducer (4.0–12.0 MHz, maximum field of view: 153 mm, maximum display depth: 160 mm) was used. Subjects were positioned supine with an extended leg in 0° and in 0° with reproducible applied valgus stress (loaded condition) through a Telos fixation device (TELOS, Wölfersheim-Berstadt, Germany) with 15 dekanewton (daN) [8]. In a second step, subjects were positioned supine with a 30° bended knee and in 30° with reproducible applied valgus stress (loaded condition) through a Telos fixation device with 15 daN (Fig. 1a, c) [8]. The medial epicondyle was palpated and the transducer was placed in the longitudinal direction. The medial femoral epicondyle and the proximal tibial plateau were used as bony landmarks, as described earlier (Fig. 2a, b) [8]. The presentation of the hyperechoic bony outline of femur and tibia has been considered as an important quality assessment for standardized measurement of medial joint width [32].

Medial knee joint width in US was measured as published by Graf et al*. *[6], Schricker et al*. *[21]*,* and Gruber et al*. *[8]*.* The distance between corresponding points on the femoral and tibial articular margins was measured in mm. All measurements were performed by a specifically trained orthopedic sports medicine resident (rater 1). Intra- and inter-rater reliability testing was conducted on 20 randomly assigned and blinded cases after an interval of six weeks by rater 1 and by one senior orthopedic surgeon (rater 2).

### Statistical analysis

Statistical analysis was performed with SPSS software (SPSS, Chicago, IL, USA) and Excel (microsoft excel 2019). For all statistical tests, *p* values less than 0.05 were considered significant.

Descriptive statistics are presented as mean ± standard deviation (SD) allowing one decimal. The Kolmogorov–Smirnov univariate normality test was used for continuous variables to confirm data normality. Since not all data were normally distributed, a table with additional specification of the median and minimum/maximum is shown in the results. Correlation between medial knee joint widening and gender was assessed using the Mann–Whitney *U* test. Correlation between medial knee joint widening in US, age, and BMI was assessed using the Pearson correlation coefficient. Medial joint space width was compared between the unloaded and the loaded states in 0° and 30° of knee flexion using paired *t* tests. Statistical correlation analysis is presented with *p* values (*p*) and the Pearson correlation coefficient (*r*) allowing three decimals.

Intra-class correlation coefficients (ICCs) were calculated to determine the intra- and inter-observer reproducibility. ICC values > 0.9 were considered excellent, values between 0.8 and 0.9 were considered good, and values < 0.8 were considered poor. An a priori power analysis based on the results of Slane et al*. *[25] and Whelan et al*.* [29] was performed with G × Power (Erdfelder, Faul, Buchner, Lang, HHU Düsseldorf, Düsseldorf, Germany). Mean medial joint width and mean change (*Δ*) between unloaded and loaded conditions and SD were calculated. The power analysis revealed a total sample size of 75 knees at an assumed effect size of 0.29 to achieve a statistical power of 0.8.

## Results

A total of 65 subjects with 79 healthy knees were enrolled in this study. None of the included subjects was secondarily excluded. 43 (54%) right knees and 36 (46%) left knees were examined. Good intra- and inter-rater reliability was observed for all US measurements. ICC values were 0.89 for unloaded and 0.89 for loaded states, respectively.

Results of US measurements are summarized in Table 1. The medial joint width did not significantly increase from unloaded to loaded conditions. The correlation between both conditions (*Δ* in 0° and *Δ* in 30°) was significant (*p* = 0.002, *r* = 0.354).

**Table 1:** Ultrasound measurements of all 79 knees: medial joint width in mm in 0° unloaded and loaded, in 30° of knee flexion unloaded and loaded, as well as the average change (*Δ*) between unloaded and loaded conditions in 0° and 30° of knee flexion

	Medial joint width (mm) in 0°	Medial joint width (mm) in 0° loaded	Medial joint width (mm) in 30°	Medial joint width (mm) in 30° loaded	Δ of medial joint width (mm) in 0°	Δ of medial joint width (mm) in 30°
Mean	5.7	7.4	6.1	7.8	1.7	1.7
Median	5.6	7.4	5.9	7.7	1.6	1.7
SD	1.2	1.4	1.1	1.2	1.0	0.9
Minimum	3.4	4.1	2.9	5.0	0.2	0.3
Maximum	9.0	10.6	8.4	11.0	4.8	4.1

*mm* millimeter, *SD* standard deviation

## Gender

In total, 32 (49%) women and 33 (51%) men were included. A significant difference between genders was evident for medial joint width in US in 0° and 30° of flexion in unloaded and loaded conditions (Table 2). Female subjects showed a lower medial joint width in unloaded and loaded conditions. There was no significant difference in the average change between unloaded and loaded conditions in 0° and 30° of knee flexion and genders.

**Table 2:** Subgroup analysis of medial joint width in US for gender (mean ± SD), in unloaded and loaded conditions, as well as the average change *(Δ*) between unloaded and loaded conditions in 0° and 30° of knee flexion

	Medial joint width (mm) in 0°	Medial joint width (mm) in 0° loaded	Medial joint width (mm) in 30°	Medial joint width (mm) in 30° loaded	Δ of medial joint width (mm) in 0°	Δ of medial joint width (mm) in 30°
Female	5.2 ± 0.9*	6.6 ± 1.1*	5.5 ± 1.0*	7.4 ± 1.0*	1.5 ± 0.9	1.7 ± 0.7
Male	6.3 ± 1.3*	8.1 ± 1.4*	6.7 ± 1.0*	8.5 ± 1.2*	1.9 ± 1.2	1.8 ± 1.1

*mm* millimeter, *SD* standard deviation

^*^p ≤ 0.05

## Age

**Fig. 3:**
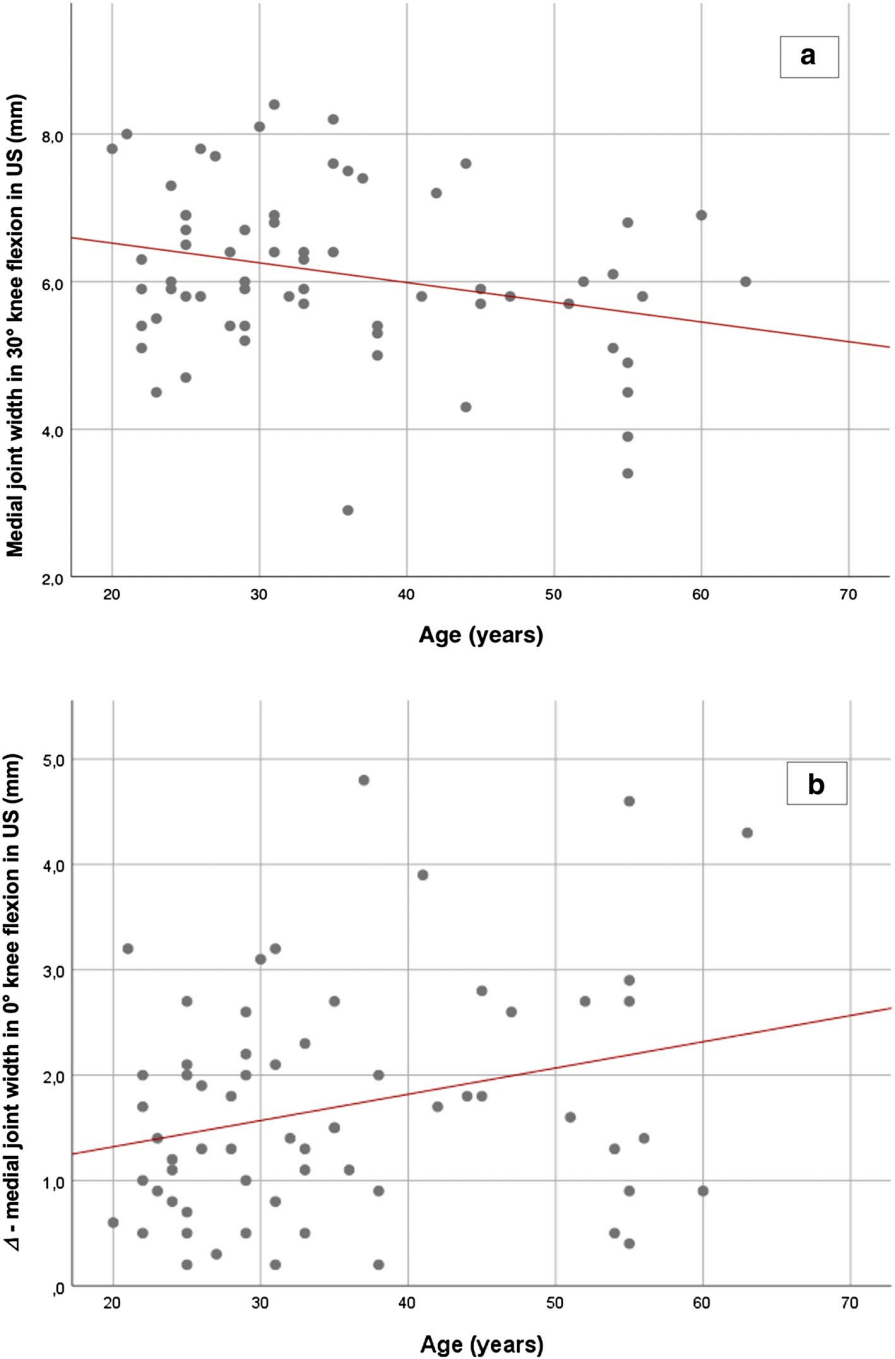
With increasing age, a significant decreased medial joint width was demonstrated in 30° of knee flexion unloaded (*p* = 0.028, *r* = 0.272, (**a**) and with increasing age, a significant increased Δ of medial joint space width was demonstrated in 0° (*p* = 0.032, *r* = 0.271, (**b**); *mm* millimeter, *US* ultrasound

The average age of enrolled patients was 35.2 ± 12.3 years (range 20–63 years). No significant correlation between medial joint width in US in 0° (unloaded and loaded), in 30° of flexion (loaded) and for the average change (between unloaded and loaded conditions) in 30° of knee flexion and age was evident. Significant correlations between medial joint width in US in 30° of knee flexion unloaded (*p* = 0.028) and for the Δ in 0° (*p* = 0.032) and age could be determined (Fig. 3a, b).

### BMI

The average BMI of enrolled patients was 25.0 ± 5.8 kg/m^2^ (range 17.2–44.8 kg/m^2^). The average BMI for women was 23.0 ± 6.1 kg/m^2^ (range 17.2–44.8 kg/m^2^) and for men 26.9 kg/m^2^ (range 20.1–42.4 kg/m^2^). No significant correlation between medial joint width in US and BMI was evident.

## Discussion

The most important finding of the present study is that medial joint width of the knee in US examination is gender-dependent. Female subjects showed a lower average medial joint width than male subjects in unloaded and loaded conditions. Also, age has a significant impact on medial joint width in various loading conditions. With increasing age, a significant increase of medial joint space width could be demonstrated between unloaded and loaded conditions in 0° of knee flexion. No significant correlation between medial joint width and BMI could be shown in this study.

Dynamic US examination of the medial structures of the knee has been proven to be a suitable method to describe medial joint space width [8, 12, 21, 25, 32]. It has been shown that the US examination is a good alternative to valgus stress radiographs, as it is radiation-free [11, 25], easy to learn [8], cheap, and allows for a dynamic assessment of the medial joint space [8, 12]. Combined with a fixation device, standardized and reproducible measurements of the medial joint width [8] are possible. In addition, new portable US devices serve as diagnostic tool and are accessible everywhere. This could be of high interest in the field of sports injuries and in outpatient clinic in follow-up examinations after non-operative or operative MCL treatment.

Few studies dealing with medial joint width in US in healthy patients have been published [8, 12, 21, 32]. Back in 1987, Schricker et al*.* first described an US-based examination of the medial joint space as diagnostic tool in medial ligament injuries of the knee [21]. However, this study was limited in its testing design including only flexed knees and no further information on how valgus stress had been performed was given.

In the present study, the examination was performed in different degrees of flexion, in a larger cohort and in a contemporary setting with modern US technologies and standardized applied valgus stress. In 1998, Gruber et al*.* combined US and standardized applied valgus stress using the Telos fixation device in their study [8]. The authors reported an average change of 2.5 ± 0.7 mm between unloaded and loaded conditions in flexed knee joints. Compared to their results, the average change between unloaded and loaded conditions was lower in our cohort. In another clinical study, Kleinbaum et al*.* not only described the average change between unloaded and loaded conditions, but also average values for medial joint space width in a small cohort of 18 healthy subjects [12]. After manually applied valgus stress, the authors described an average increase of the medial joint space width to 9.6 mm from 6.7 mm in a neutral position. It has to be noted, however, that the authors did not specify the degree of flexion in this investigation and that the cohort was small. In contrast to the results of the present study, Slane et al*.* reported a significant increase of the medial space width from unloaded to loaded conditions in a cadaveric setting [25]. The average change between unloaded and loaded conditions in approximately 20° of knee flexion in this cadaveric study (10 knee joints, load: 10 Nm, US examination) has been reasonably similar to our results [25].

There exist several studies dealing with medial joint space width in healthy knees, in which the examiners used radiological methods where ionizing radiation was applied [13, 25, 29].

LaPrade et al*.* investigated the correlation of valgus stress radiographs with medial knee ligament injuries in a biomechanical setting [13]. They described a mean medial joint width of 6.9 ± 0.8 mm in 0° of knee flexion in loaded condition (10 Nm). In 20° of knee flexion, the mean medial joint width was 6.4 ± 0.9 mm (loaded). When they used 15 or 20 Nm of load, forces produced premature medial structure failure of the specimens and the knee could not be used for further testing [13]. Compared to this, in a greater degree of knee flexion, medial joint space width increased in our study.

Previous cadaveric studies using fluoroscopic imaging to describe the medial joint space indicate a greater average medial joint space width in loaded conditions [25, 29]. Reasons for those variations remain unknown; possible explanations are differences in applied load or individual differences in biomechanical settings.

A recently published study of Zhu et al*.* found higher joint space widths in male subjects [32].

Similarly, in the present investigation, female subjects showed a lower average medial joint width than male subjects in unloaded and loaded conditions. Although women tend to have a greater knee laxity than men concerning the ACL [18], we could not confirm this fact in case of the MCL, since the average change between unloaded and loaded conditions did not differ between genders in the present study.

Also, age had a significant impact on medial joint width, especially concerning the change between unloaded and loaded conditions in 0° of knee flexion. This fact could be due to an age-dependent relative reduction of the medial joint space through increased cartilage wear and simultaneous capsule shortening due to osteoarthritis [17, 32].

Several limitations of the current study have to be mentioned. First, US is a highly operator-dependent imaging method. Second, pain-related muscle contraction of the patient during the dynamic examination of the knee joint in valgus stress can lead to a change of results. Therefore, the patient’s state of relaxation was controlled through direct physical contact between patient and examiner [8]. Third, the US examination has not been correlated with comparable clinical or other radiological methods, but in a previous study, similar values for measures of absolute medial joint space width between US and fluoroscopic imaging could be demonstrated [25]. Fourth, due to ethical reasons, limb alignment in healthy subjects was only clinically estimated using the Caliper method [9]. Additionally, side-to-side differences could not be demonstrated in this study, as only the results of healthy knees have been considered.

The results of this study have clinical relevance when considering diagnosis of frequent ligamentous injuries to the medial side of the knee. Particularly, results of standardized US measurements with and without applied valgus stress in a healthy cohort were described. These findings of US evaluation of the medial joint width could help clinicians to better diagnose and classify acute or chronic MCL injuries. Thus, it might simplify follow-up examinations of non-operative or operative MCL treatment in outpatient clinic and to estimate medial laxity in preventive training and return to sports. Furthermore, dynamic US examinations of the medial structures of the knee could replace valgus stress radiographs and fluoroscopic imaging in the future.

## Conclusion

In conclusion, the present study demonstrated that gender and age affect medial joint space width in healthy knees. Ultrasound has turned out to be a suitable tool to assess laxity of the medial collateral ligament complex dynamically and allows for a fast location-independent evaluation of the function without ionizing radiation. Present results might be helpful to enhance clinical examination of the MCL.
